# Impact of COVID-19 pandemic on socioeconomic and mental health aspects in Nepal

**DOI:** 10.1177/0020764020942247

**Published:** 2020-07-10

**Authors:** Kritika Poudel, Pramod Subedi

**Affiliations:** 1 Graduate School of Health Sciences, Hokkaido University, Sapporo, Japan; 2 Department of Biochemistry and Genetics, La Trobe Institute for Molecular Science, La Trobe University, Melbourne, VIC, Australia

**Keywords:** COVID-19, psychosocial, socioeconomic, mental health

## Abstract

**Background:**

Public health concern is increasing with recent rise in the number of COVID-19 cases in Nepal. To curb this pandemic, Nepal is facing some forms of lockdown, encouraging people to implement social distancing so as to reduce interactions between people which could eventually reduce the possibilities of new infection; however, it has affected the overall physical, mental, social and spiritual health of the people.

**Methods:**

Published articles related to psychosocial effects due to COVID-19 and other outbreaks were searched and reviewed.

**Conclusion:**

While many countries are supporting their citizens with sophisticated health safety-nets and various relief funds, some developing countries have unique challenges with vulnerable populations and limited resources to respond to the pandemic. This review presents the consequences of pandemic and lockdown on socioeconomic, mental health and other aspects in Nepalese society.

## Introduction

A novel coronavirus named severe acute respiratory coronavirus 2 (SARS-CoV-2) was first identified in a seafood market in Wuhan City, Hubei Province in China, at the end of 2019 (Zhu et al., 2020). The contagious respiratory illness caused by this novel coronavirus is called coronavirus disease 2019 or, in short, COVID-19 (Wu et al., 2020). From February, COVID-19 cases soared across most of Europe, the United States, Australasia, Asia and on to Africa. Until now, the novel coronavirus continues to wreak havoc on daily life around the globe, affecting 213 countries, infecting 8,018,963 people and killing 436,138 people (until 15 June 2020; Worldometer, 2020).

**Figure 1.**
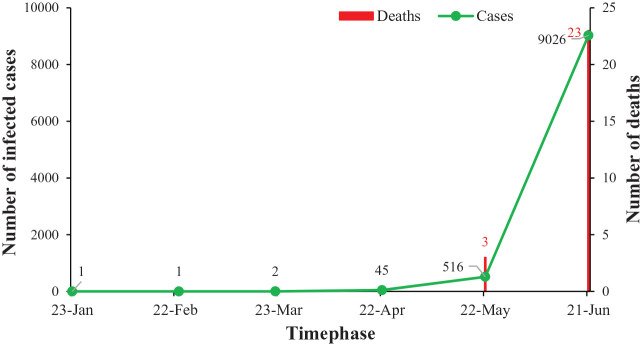
Number of COVID-19 infected cases and deaths in Nepal (as per 21 June 2020).

On 13 January, a 31-year-old Nepali student of Wuhan University, who had returned home on 5 January, was admitted with mild symptoms (Bastola et al., 2020). He got discharged on 17 January after preliminary tests showed he may not be infected. The public laboratories in Nepal did not have reagents required for testing and there were no suspected cases needing testing. Hence the samples were sent to Hong Kong for testing, which showed positive results for COVID-19 (*The Kathmandu Post*, 2020d). This was the first ever reported case in South Asia (NDTV, 2020). With no new case reported in February, a second case of COVID-19 was seen on 23 March, a 19-year-old woman who had returned from France on 17 March (*The Kathmandu Post*, 2020c). With a slow start, the total confirmed cases reached to 57 on 30 April. By the end of May, the total number of confirmed cases nationwide reached 1,567. Until this article was prepared (21 June), more than 9,026 and overall 74 districts have been tested positive for the novel coronavirus resulting in different physical, socioeconomic and psychological impacts on the Nepalese (Ministry of Health and Population Nepal, 2020). Figure 1 shows the number of COVID-19 infected cases and deaths in Nepal (as per 21 June 2020).

## Impact of COVID-19 lockdown

Lockdown is considered to be an effective measure in slowing the spread of coronavirus around the globe (Barkur et al., 2020; Flaxman et al., 2020). To further stop the spread of the virus, many countries are currently in some degree of lockdown. Until then, extreme social distancing is pretty much the only intervention available to keep healthy individuals spaced from each other. Even in the best-case scenario, coronavirus vaccine development is likely to take 12–18 months (*The New York Times*, 2020).

**Figure 2.**
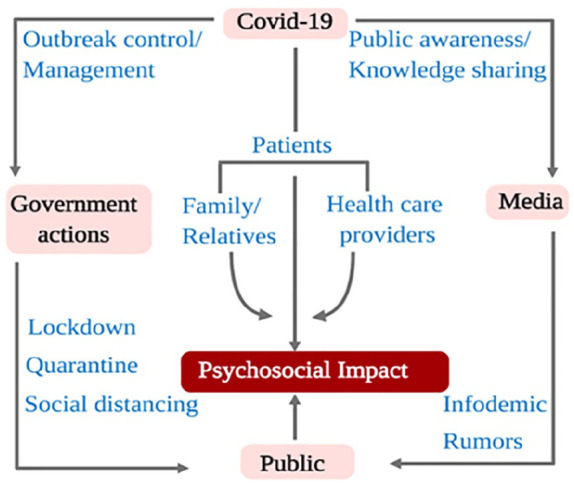
Psychosocial relationship among COVID-19, media, government actions and the public.

While the preventive vaccine and treatment option are yet to be developed, the worldwide spread of the novel coronavirus has further led to neuropsychiatric issues such as fear, anxiety, depression, panic attacks, psycho-motor excitement, suicidal deaths and a general decrease in overall wellbeing (Brooks et al., 2020; Xiang et al., 2020). Similarly, patients who are infected with COVID-19 are at a greater risk of developing mental health problems, as they are facing stigma and discrimination from their own family members. Similar situations were faced by the general public as well as many medical practitioners during previous outbreaks such as Severe Acute Respiratory Syndrome (SARS), Middle East Respiratory Syndrome (MERS) and Ebola (Jeong et al., 2016; Leary et al., 2018; Rogers et al., 2020; Rubin & Wessely, 2020; Wing & Leung, 2012). Until now, there is a paucity of information on the socioeconomic and psychological aspects of the Nepalese community in the face of COVID-19, which is critical for guiding policies and interventions to curb the pandemic. Figure 2 shows the psychosocial relationship among COVID-19, media, government actions and the public.

### Impact on trade and tourism

Being a landlocked country in South Asia, India and China are the major trade countries for Nepal. As the precautionary measures to avoid COVID-19 spread in Nepal, the Government of Nepal closed the Rasuwagadhi border on 28 January, completely halting Nepal–China trade (*My republica*, 2020a). This closure limited the availability of raw materials which used to come from China for manufacturing goods. On 22 March, Nepal closed its open borders with India, resulting in serious effects in the import and export of goods (*The Economic Times*, 2020). This resulted in a situation of panic-buying and hoarding of goods among the Nepalese, creating a shortage of goods and supplies. Thousands of Nepalese, who wanted to return to Nepal got stuck at Indian border points (*Al Jazeera*, 2020). The travel restrictions to and from different international destinations was put into action as a measure to prevent the spread of COVID-19 infection, which massively affected the tourism sector. With the implications of countrywide lockdown on 24 March, the international flight ban resulted in the stranding of thousands of Nepalese in different parts of the world. A large number of Nepalese labor migrants and students could not return to their destinations abroad from Nepal, resulting in serious havoc.

With the World Health Organization (WHO) encouraging people to wash hands with soap and water or an alcohol-based hand sanitizer, and proper usage of facial masks for providing protection against spreading of the coronavirus, it ignited panic-buying and hoarding of these goods, leading to shortages in the majority of cities in Nepal (*The Kathmandu Post*, 2020a; World Health Organization, 2020a). As Nepal heavily relies on China and India for protective gear such as face masks, gloves and caps, the export ban from these countries has created a huge scarcity in supplies and deliveries, leading to mental stress among health care workers, hygienic staff and the public. To deal with this crisis, some hospitals started sewing masks using plain cloth, however, the shortage of proper personal protective equipment (PPE) continues (*The Kathmandu Post*, 2020b).

Due to the COVID-19 outbreak, the hardest hit sector of the economy is tourism. The World Travel and Tourism Council research reported that Nepal’s tourism sector was responsible to generate Rs. 240.7 billion in revenue and supported more than 1.05 million jobs in 2018 (*The Kathmandu Post*, 2019). Postponing of Visit Nepal 2020, and suspension of on-arrival visas along with countrywide lockdown, has led to the loss of thousands of jobs. In all, 20,000 tour, trek and guides, and porters lost their livelihood when mountaineering was suspended (*The Kathmandu Post*, 2020f). With high levels of food insecurity and widespread malnutrition among children, the consequences of the virus spreading widely could reverse the recent positive trends in poverty and prove to be catastrophic and far-reaching. Nepal, being the fifth most remittance-dependent country, sends millions of labor migrants abroad every year for international labors. While remittances range up to 25% of the gross domestic product (GDP), migrant remittances may decline during the time of COVID-19, limiting the source of income of households in Nepal (World Bank, 2020a). The declination in remittance can limit the families from getting out of poverty, paying off unscrupulous loans, and investment in education, health and land.

### Impact on agriculture

The agricultural crops, livestock and fisheries are not outside the impact of COVID-19. Being an agricultural country, the travel restriction and lockdown have affected every stage of the food supply chain, including food production and distribution in Nepal. Farmers are compelled to dump milk and vegetables after a significant decrease in supply and closure of processing companies and proper markets (Poudel et al., 2020). This has led to sudden price hike, black marketing and shortage of products in the local markets. While the government is urging people to follow quarantining and limiting gathering of people, millions of farmers have to gather together to sow food and cash crops in Nepal with the arrival of the monsoon. The current lockdown measures might help the government to defeat this virus, but if the patterns of small-scale planting, harvesting and distribution continue to be disturbed, hundreds of thousands will lose their livelihoods, and the whole nation could slip into deep food insecurity. Therefore, the government should enforce measures to control the pandemic without disturbing the food supply chain and considering the food security of their citizens.

### Impact on education

The action of the government of Nepal to close all educational institutions, postponing of all national level examinations and prohibiting the gathering of more than 25 people together led to an outflux of more than 300,000 people from Kathmandu in 3 days (*Rising Nepal Daily*, 2020). Perceiving the village environment as pure, free from germs and contamination, and unlikely to get coronavirus might have led to the surge in the outflux of people. The drastic increase in new infection rates, lesser tests, increased media reporting and death tolls have increased public anxiety. The absence of clear messages and the desire for facts have heightened fear among the public and propelled them to seek information from less reliable portals (Rubin & Wessely, 2020). The current pandemic has imposed multiple restrictions on research as laboratories have been closed, and scientists and researchers have been working from home, limiting recruitment in studies.

According to the World Bank (2020b), the COVID-19 pandemic has caused more than 1.6 billion children and youth in 161 countries to be out of school, which is close to 80% of the world’s enrolled students. Parents have experienced increased pressure to work from home, to keep their work running as well as to take care of schooling children at home at the same time, while caregiver resources including grandparents and the wider family have been restricted (Fegert et al., 2020). With the unprecedented lockdown, most parents have worries about their children’s education and future as their school education has been halted until further notice. The Government of Nepal though has decided to introduce a digital education system to continue the teaching learning process, and this has further burdened parents with the load of school fees and online internet fees. It is further stressful for parents with a low income who have to struggle for daily wages and do not have proper internet access, as it compromises the learning needs of their children. While Nepal can boost inexpensive, accessible and familiar resources such as radio networks, television broadcasting and social media for remote learning, it is important to assess the sustainability of its own existing resources, and checking the possibilities before deliberately imposing them on academic institutions and the public.

### Impact on media sectors

The infodemics, misinformation and inaccurate conception are spreading quicker from fake and unauthorized news portal websites, contributing to myths and rumors in the society (Figure 1). Myths related to alcohol, adding hot peppers, ginger and garlic to food, and exposing oneself to temperatures higher than 25° or to cold weather and snow to kill the coronavirus are misleading people. Therefore, there is a need to be thoughtful and conscious when communicating on social media and other communication platforms (UNICEF, 2020a). Authorized health organizations and the government should provide timely information through reliable portal platforms and ban unauthorized websites to avoid misleading the public.

A large number of people have assumed the lockdown as vacation time and are pressurizing others to engage in forceful academic or job-related activities. During the lockdown, several social networking media messages, instead of promoting, are actually compromising the mental health of individuals in the society. People have their individual coping strategies and not all can perceive the pandemic lockdown as an opportunity to learn. Several messages are demanding people to come out with new skills during the lockdown, which has further aggravated the psychological pressure and mental stress. This pressure has provoked regret, shame, overwhelming feelings and negative thoughts along with decreased self-esteem, which should be addressed immediately to avoid mental breakdown (Mukhtar, 2020).

### Impact on health

This pandemic crisis has significantly transformed the working environment, resulting in high-pressure work, and unfavorable and demanding interactions among health workers. Frontline health workers, including doctors, nurses, certified caregivers, lab technologists and pharmacists, with inadequate supplies of PPE, have been giving their best professional services to protect human lives. While trying to balance life as a healthcare professional and as a member of a family, dealing with highly infectious clients has led to guilt about potentially exposing their families to infection (Ramaci et al., 2020).

Contracting COVID-19 has increased stigma and social discrimination among people. Some house owners have been reported to evict nurses, doctors and other medical professionals from their rental apartments fearing the spread of the novel coronavirus in their neighborhood. Cured patients upon returning home are socially avoided and discriminated against, leading to decrease in moral support. Stigma can negatively affect clients searching for medical care at a time when they are at their most vulnerable stage. Stigma and social discrimination can lead to hiding of symptoms and avoid seeking of medical care, making it tremendously difficult for health care professionals and the government to control the disease. This stigmatization can discourage people from adopting healthy behaviors and can dramatically increase the suffering of people, leading to fatigue, stress and other mental distress. Hence, by understanding the disease, building trust, showing empathy to those affected, and adopting effective practical measures, people can help to save their dear ones (UNICEF, 2020b).

The COVID-19 pandemic has brought serious psychological impact among health workers, students and the general public around the globe, as presented in Table 1.

**Table 1.** Studies reporting psychological impacts due to COVID-19 pandemic.

Country	Participants	Sample size	Results	References
China	Health workers	2,182	Insomnia, anxiety, depression, somatization and obsessive-compulsive symptoms	Zhang et al. (2020)
America	Mount Sinai Health System (MSHS) employee, faculty, and trainee crisis support, task force	NA	Fear for their personal safety	Ripp et al. (2020)
China	General public	1,210	Higher levels of stress, anxiety and depression	Wang et al. (2020)
China	Students	2,330	Depressive symptoms, including anxiety	Xie et al. (2020)
China	Healthcare workers	1,257	Depression, anxiety, insomnia and distress	Lai et al. (2020)
India	General public	1,106	Significant psychological impact	Varshney et al. (2020)
China	Medical staff treating patients with COVID-19	180	Anxiety, negativity, poor sleep quality and self-efficiency social support	Xiao et al. (2020)

Although Nepal lacks routine national-level data on suicide, media together with police data suggest that about 5,000 Nepalese commit suicide every year. In addition, a significant rise in the number of people committing suicide was observed in earthquake-exposed populations. This trend appears to be a familiar pattern across the world; a notable increase in suicide rates occur after natural disasters such as floods, hurricanes and pandemic (Goldmann & Galea, 2014). The pandemic-related restraints, such as spatial distancing, isolation and home quarantine, are impacting on economic sustainability and wellbeing, which may induce psychological mediators such as sadness, worry, fear, anger, annoyance, frustration, guilt, helplessness, loneliness and nervousness (Bhuiyan et al., 2020; Mukhtar, 2020). In the first month of the nationwide lockdown, a total of 487 people committed suicide, which is 20% more compared with mid-February to mid-March, when the number stood at 410 (*My Republica*, 2020b). The data compiled by Nepal Police shows that, from the onset of lockdown, 23 March till 6 June, a total of 1,227 suicide (16.5 a day) cases were filed, which is seriously high compared with the total of 5,785 people (15.8 a day) in the previous year (*The Kathmandu Post*, 2020e). The deceased had used burning, stabbing, drowning, jumping from heights as major ways of attempting suicides; while the exact cause of death remains unknown, psychiatrists have linked it with the mental health of people who have been forced to stay indoors during lockdown. The sudden economic recession, unemployment, poverty, social isolation and economic distress might lead individuals to contemplate suicide. While Nepal faces a total of 23 deaths (until 21 June) due to COVID-19, the suicidal rate is higher by several folds, alarming the mental health status of people and the sudden need to address them. As self-harm is predominantly found among adolescents, an upsurge of self-injurious and suicidal behavior in the youth requires urgent attention (Fegert et al., 2020). The government and nongovernment agencies are running several helplines to provide mental health counseling over the phone; however, as the risks to mental health rise, there is urgent need to increase investment in mental health services. The WHO has already predicted the rise in the number of mental health problems due to the global pandemic; during this time, the government should focus on addressing the mental health wellbeing of the Nepalese people.

### Impact on vulnerable people

According to the Nepal Living Standards Survey 2010–2011, 25% of the population lived below the poverty line (Central Bureau of Statistics, 2011). The link between poverty and communicable disease is well-evident (Alsan et al., 2011; Bhutta et al., 2014). COVID-19 is no exception and has triggered increasing unemployment, loan defaults and major economic losses around the globe (Kantamneni, 2020). The economic downturn caused by COVID-19 can increase the economic instability, health inequalities and social disparities in Nepal, which can have a huge impact on the poverty levels. While the lockdown has affected traders, especially people with small shops and those with limited sources of income, the poor, marginalized people and daily wagers are more vulnerable. Research has shown that a pandemic like COVID-19 can result in increased mental burden to marginalized or low-income people via socioeconomic disadvantage such as job insecurity, housing instability, discrimination and food insecurity (Goldmann & Galea, 2014).

As per the Nepal Census, nearly 2% of the total population of Nepal is reported to have some kind of disability (UNFPA Nepal, 2020). Compared with persons without disabilities, disable people are generally more likely to have poor health and are therefore more vulnerable to deficiencies in health care services. Many persons with disabilities do have specific underlying conditions that make a disease like COVID-19 more dangerous for them. The limited access to culturally respected information, personal assistance and medical care has impacted persons with disabilities from minority communities. However, lack of local government coordination with organizations persons with disabilities and local community leaders and bureaucratic barriers has prevented this group from being counted and included in relief efforts, which can result in starvation, and prevention of passing on intergenerational knowledge (Minority Rights Group International, 2020).

## Conclusion

The lockdown curfews, self-isolation, social distancing and quarantine have affected the overall physical, mental, spiritual and social wellbeing of the Nepalese. With the beginning of lockdown, the government decided to shut down all cinema halls, gyms, health clubs and museums, as well as banned the gathering of people for cultural, social or religious activities, including temples, monasteries, churches and mosques. In the case of death, the pandemic has disrupted the normal bereavement processes of families. Although these measures are taken for the protection of people from COVID-19, it has created fear, anxiety and uncertainty among the Nepalese, which needs to be addressed immediately. The economic recessions have put significant financial pressure on many families, which might increase unhealthy conflict, family breakdown, abuse, depression and domestic violence. The psychological impacts of the COVID-19 lockdown might be a challenge for an indefinite time, hence it is necessary to emphasize and address coping strategies, mental health interventions and awareness using the available resources. To deal with the current pandemic and future health emergencies, the government should be equipped with adequate health logistics, technologies and skilled manpower, and needs to develop its capacity in health financing to foresee future opportunities and challenges. By strengthening the health care workforce, conducting mandatory health education and training in schools, wisely utilizing existing health manpower, investing and expanding the scope of health research and establishing well-equipped laboratories, Nepal needs to be prepared for re-emergence or probably another outbreak. The long-term battle with coronavirus may help people to win against it by developing vaccines and medicines; however, in the long run, the country must be prepared for numerous mental health threats associated with the pandemic and actions should be established and implemented.
